# Laparoscopic resection of neuroblastomas in low- to high-risk patients without image-defined risk factors is safe and feasible

**DOI:** 10.1186/s12887-017-0826-8

**Published:** 2017-03-14

**Authors:** Chiyoe Shirota, Takahisa Tainaka, Hiroo Uchida, Akinari Hinoki, Kosuke Chiba, Yujiro Tanaka

**Affiliations:** Department of Pediatric Surgery, Nagoya University Graduate School of Medicine, Nagoya, Japan

**Keywords:** Image-defined risk factors, Minimally invasive surgery, Laparoscopy, Neuroblastoma

## Abstract

**Background:**

Several studies have reported that minimally invasive surgery (MIS) might be considered for resecting neuroblastomas without image-defined risk factors (IDRFs); however, there are no studies comparing the outcomes of laparotomy and laparoscopy in IDRF-negative patients. Thus, we investigated the feasibility of laparoscopic surgery and compared the two abovementioned approaches.

**Methods:**

To compare the effects of laparotomy with those of laparoscopy in patients with neuroblastomas without IDRFs, the following items were retrospectively compared: largest tumor dimension, volume of blood loss, time required to initiate postoperative feeding, locoregional recurrence rate, survival, etc.

**Results:**

Nine patients without IDRFs (three at low-to-medium risk and six at high risk) underwent laparotomy, and seven patients without IDRFs (two at low-to-medium risk and five at high risk) underwent laparoscopy. Median duration of surgery was 221 (130–304) and 172 (122–253) min in the laparotomy and laparoscopy groups, respectively, showing no significant difference. Median postoperative time required for resuming meal consumption was significantly longer in the laparotomy (4 days; 2–5) group than that in the laparoscopy group (3 days; 2–3; *p* = 0.023). Median blood loss was significantly higher in the laparotomy group (5 ml/Kg;2.6–16) than that in the laparoscopy group (2.1 ml/Kg;0.1–4.0; *P* = 0.037). Median follow-up period was 81 (52–94) and 21 (17–28) months, locoregional recurrence rates were 22 and 0% at 1 year, 1-year progression-free survival rates were 78 and 100%, and overall survival rates were 67 and 100% in the laparotomy and laparoscopy groups, respectively, with no significant differences.

**Conclusions:**

MIS for the treatment of neuroblastomas without IDRFs in low- to high-risk patients is safe and feasible and does not compromise the treatment outcome.

## Background

Neuroblastoma is a common malignant solid tumor in pediatric cases that is usually treated by a combination of observation, surgery, chemotherapy and radiation therapy. Surgical resection is a part of the multidisciplinary approach, for which it is important to decrease the general invasiveness and damage to the surrounding tissues and promptly transition to subsequent treatments [1–3]. Image-defined risk factors (IDRFs) are surgical risk factors that are evaluated according to the concepts established by the International Neuroblastoma Risk Group, as proposed by collaborative studies conducted in Europe [4, 5]. The significance of preoperative evaluation of surgical risks by internationally integrated standards that are made possible by IDRFs is remarkable, and the establishment of standardized criteria for and assessment of not only the advisability of surgery but also the selection of surgical procedure is expected. The prevalence of clinical studies on surgery using IDRFs is anticipated to increase in future.

Compared with laparotomy, laparoscopic surgery is generally associated with rapid recovery and less invasiveness [6, 7]. Laparoscopic surgical resection for selected pediatric malignant solid tumors was found to be both feasible and safe [8, 9]. Long-term follow-up data are essential to confirm its oncologic safety [10], and several reports have claimed that minimally invasive surgery (MIS) might be considered to resect neuroblastomas without IDRFs [11, 12]. Minimally invasive adrenalectomy is a promising approach for the resection of small adrenal tumors, and benign diseases are excellent candidates for this minimally invasive technique [13]. However, there have been no studies comparing laparotomy and laparoscopy for the treatment of neuroblastomas in low- to high-risk patients without IDRFs. Thus, we investigated the feasibility of laparoscopic surgery and attempted to compare both these approaches in IDRF-negative patients.

## Methods

Prior to this study, all protocols were approved by the ethics review board of our institute (approval number: 2015–0086). The medical records of all patients aged 0–15 years who underwent total or partial resection of abdominal neuroblastoma at our department between August 2003 and August 2016 were retrospectively reviewed. Laparoscopic resection was introduced in February 2014 and was chosen when the tumor had no IDRF-positive factor except contact with renal vessels.

Determination of the therapeutic strategy was based on the Japan Neuroblastoma Study Group (JNBSG) protocol, where surgical intervention was considered when the patients achieved IDRF-negative status and had no metastatic lesions [14–16]. To evaluate the effects of laparoscopic surgery, patients with neuroblastomas without IDRFs who underwent laparotomy were compared with those who underwent laparoscopy. The following items were retrospectively compared: N-myc proto-oncogene protein (MYCN), vanillylmandelic acid (VMA), homovanillic acid (HVA), neuron-specific enolase (NSE), largest tumor dimension, duration of surgery, volume of blood loss, time required to initiate postoperative feeding, locoregional recurrence rate and survival.

Statistical analysis was performed using the Fisher’s exact test for categorical variables and Wilcoxon’s signed-ranktest for continuous variables with JUMP pro ® 11 (SAS Institute Inc., Cary, NC, USA). A *p*-value of <0.05 was considered statistically significant.

### Surgical laparoscopic procedure

The patient was placed in the supine or semi-lateral decubitus position, three flaps were created in the umbilicus in an inverted Y shape, a multi-port device was inserted, and the first port was placed [17]. Through the multi-port device, one or two additional ports and one or two working ports were inserted in the abdomen, and abdominal air pressure of 8–10 mmHg was applied. Resection was performed using laparoscopic coagulating shears. Then, the resected tumor was placed in a specimen retrieval bag and removed through the umbilical layer if it was 5 cm or less in diameter or by performing an additional Pfannenstiel incision if larger.

## Results

Thirty-four patients (six at low-to-medium risk and 28 at high risk) underwent laparotomy, and nine patients (two at low-to-medium risk and seven at high-risk) underwent laparoscopy. The proportion of IDRF-negative patients in the laparoscopy group was significantly greater than that in the laparotomy group (78% vs. 26%; *p* = 0.008). No differences in sex, age, MYCN, VMA, HVA, NSE or tumor size (largest tumor dimension) were apparent between the groups. Only one of the two IDRF positive case was converted to laparotomy (Table 1).

**Table 1:** Resection of abdominal neuroblastoma performed between August 2003 and 2016

	Laparotomy (*n* = 34)		Laparoscopy (*n* = 9)		*p*
			Total laparoscopic resection 8 (89%)	Conversion 1 (11%)	
Removal	Total 18 (53%)	Partial 16 (47%)	Total 8 (100%)	Partial 1 (100%)	
Age (months) ^a^	45 [12–60]		28 [21.5–51]		0.300
Sex	males 12 (35%)	females 22 (65%)	males 5 (56%)	females 4 (44%)	0.440
IDRF (%)	IDRF+ 25 (74%)	IDRF− 9 (26%)	IDRF+ 2 (22%)	IDRF− 7 (78%)	***0.008*** ^b^
MYCN amplified	amplified 9 (26%), single 14 (41%), unknown 11 (32%)		amplified 2 (22%), single 3 (33%), unknown 4 (44%)		0.790
HVA (mg/gCr) ^a^	16.1 [13.1–23.8]		18.2 [11.2–43.3]		0.702
VMA (mg/gCr) ^a^	9.70 [8.9–17.8]		9.1 [4.0–24.7]		0.702
NSE (ng/mL) ^a^	11.4 [9.0–15.7]		19.1 [11.0–21.0]		0.051
Largest tumor dimension (cm) ^a^	4.0 [3.0–6.0]		4.3 [2.6–4.8]		0.704

*IDRF* image-defined risk factor, *MYCN* N-myc proto-oncogene protein, *VMA* vanillylmandelic acid, *HVA* homovanillic acid, *NSE* neuron-specific enolase

^a^Median [Interquartile range]

^b^This *p* value shows that the proportion of IDRF-negative patients in the laparoscopy group was significantly greater than that in the laparotomy group

**Fig. 1:**
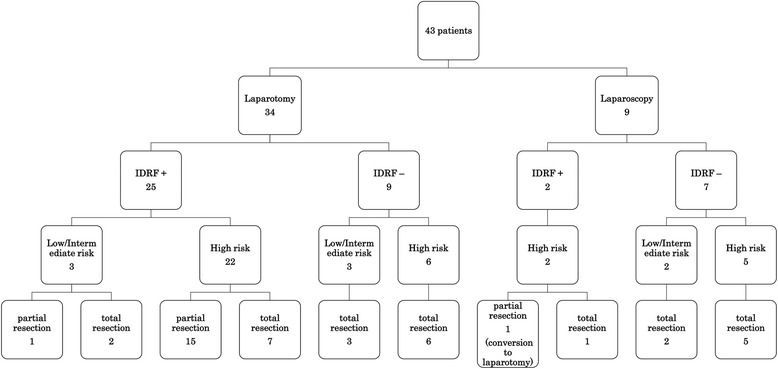
Patient and risk-group stratifications

In the laparotomy group, total resection was performed in nine out of 25 IDRF-positive patients; partial resection was performed in the remaining 16 (Fig. 1).

**Fig. 2:**
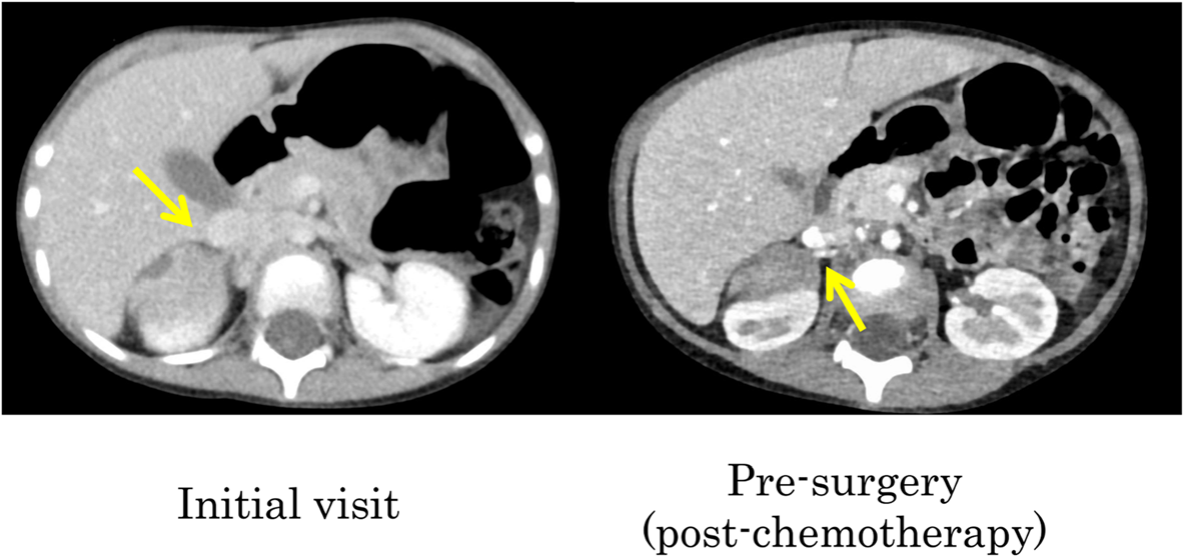
A 51-month-old girl (International Neuroblastoma Staging System [INSS]: stage 4, Children’s Oncology Group [COG] risk: high, surgical duration: 202 min, and blood loss: 3 mL). Total resection was laparoscopically performed. On her first visit, imaging studies showed that the renal vessels and aorta were encased by the tumor. After neoadjuvant chemotherapy, the tumor was seen to be in contact with the right renal vessels only. Outcome: progression-free survival, 23 months

In the laparoscopic group, one of two IDRF-positive patients was converted from laparoscopy to laparotomy during the course of the procedure; dissection was difficult because of strong adhesions around the renal vessels. IDRF was positive in this case, and the renal artery was encased by the tumor so he underwent laparotomy with partial resection. The other IDRF-positive patient underwent laparoscopy with total resection (Fig. 2).

Nine IDRF-negative patients (three at low-to-medium risk and six at high risk) underwent laparotomy, and seven IDRF-negative patients (two at low-to-medium risk and five at high risk) underwent laparoscopy (Fig. 1), and the outcomes were compared between the two groups. Seven of the 9 cases in the laparotomy group and 5 of the 7 cases in the laparoscopy group received neoadjuvant chemotherapy, consisting of vincristine, cyclophosphamide, ifosfamide, doxorubicin, pirarubicin, cisplatin, carboplatin and etoposide, based on the JNBSG risk-matched multidrug regimen. Total resection was performed in all patients. There were apparent differences in age, sex, body weight, Kaup index, MYCN, VMA, HVA, NSE and tumor size (largest tumor dimension) between the groups. The following items were compared between IDRF-negative patients who underwent laparotomy (*n* = 9) or laparoscopy (*n* = 7): duration of surgery, volume of blood loss, time required for initiating postoperative meal consumption, locoregional recurrence at 1 year, and survival. The median duration of surgery was 221 min (130–304) in the laparotomy group and 172 min (122–253; *p* = 0.626) in the laparoscopy group, indicating no significant difference. The median duration to resume meal consumption was significantly longer in the laparotomy group (4 days; 2–5) than that in the laparoscopy group (3 days; 2–3; *p* = 0.023). The median volume of blood loss was significantly higher in the laparotomy group (5 mL/kg body weight; 2.6–16) than that in the laparoscopy group (2.1 mL/kg body weight; 0.1–4.0; *p* = 0.037). The median follow-up period was 81 and 21 months, locoregional recurrence rates at 1 year were 22% (2 of 9 cases) and 0%, 1-year progression-free survival rates were 78 and 100%, and overall survival rates were 67 and 100% in the laparotomy and laparoscopy groups, respectively, indicating no significant differences (Table 2). No surgical complications (greater than Grade II severity, according to the Dindo classification) were observed in the IDRF-negative group [18].

**Table 2:** Characteristics of IDRF-negative patients who underwent laparotomy or laparoscopic tumor resection

	Laparotomy (*n* = 9)		Laparoscopic surgery (*n* = 7)		*p*
Risk	Low/intermediate 3 (33%)	High 6 (67%)	Low/intermediate 2 (29%)	High 5 (71%)	0.838
Surgery	total removal 9 (100%)		total removal 7 (100%)		
Age (month) ^a^	12 [9.5–48]		28 [23–51]		0.143
Sex	male 6 (67%)	female 3 (33%)	male 3 (43%)	female 4 (57%)	0.615
Body weight (kg) ^a^	8.65 [8.09–15.2]		11.8 [10.3–14.9]		0.143
Kaup index ^a^	15.32 [13.87–16.65]		15.67 [15.51–16.45]		0.626
MYCN amplified	amplified 2 (22%), single copy 2 (22%), unknown 5 (56%)		amplified 2 (29%), single copy 2 (29%), unknown 3 (43%)		0.771
VMA (mg/gCr) ^a^	11.1 [9.6–17.0]		9.1 [7.8–19.8]		0.805
HVA (mg/gCr) ^a^	21 [17.7–30.3]		18.2 [11.2–30.4]		0.805
NSE (ng/mL) ^a^	9.6 [8.0–29.4]		19.3 [12.4–21.0]		0.326
Largest tumor dimension at surgery (cm) ^a^	3.3 [2.9–4.1]		3.6 [2.7–5.0]		0.626
Duration of surgery (min) ^a^	221 [130–304]		172 [122–253]		0.626
**Loss of blood volume (mL/kg)** ^a^	**5 [2.6–16]**		**2.1 [0.1–4.0]**		***0.037*** ^b^
**Time to start postoperative feeding (days)** ^a^	**4 [2–5]**		**3 [2–3]**		***0.023*** ^b^
Locoregional recurrence (1 year)	22%		0%		0.475
Progression-free survival (1 year)	78%		100%		0.414
Overall survival (1 year)	67%		100%		0.231
Follow up (months) ^a^	81 [52–94]		21 [17–28]		

*IDRF* image-defined risk factor, *MYCN* N-myc proto-oncogene protein, *VMA* vanillylmandelic acid, *HVA* homovanillic acid, *NSE* neuron-specific enolase

^a^Median [Interquartile range] ^b^Significant differences

**Fig. 3:**
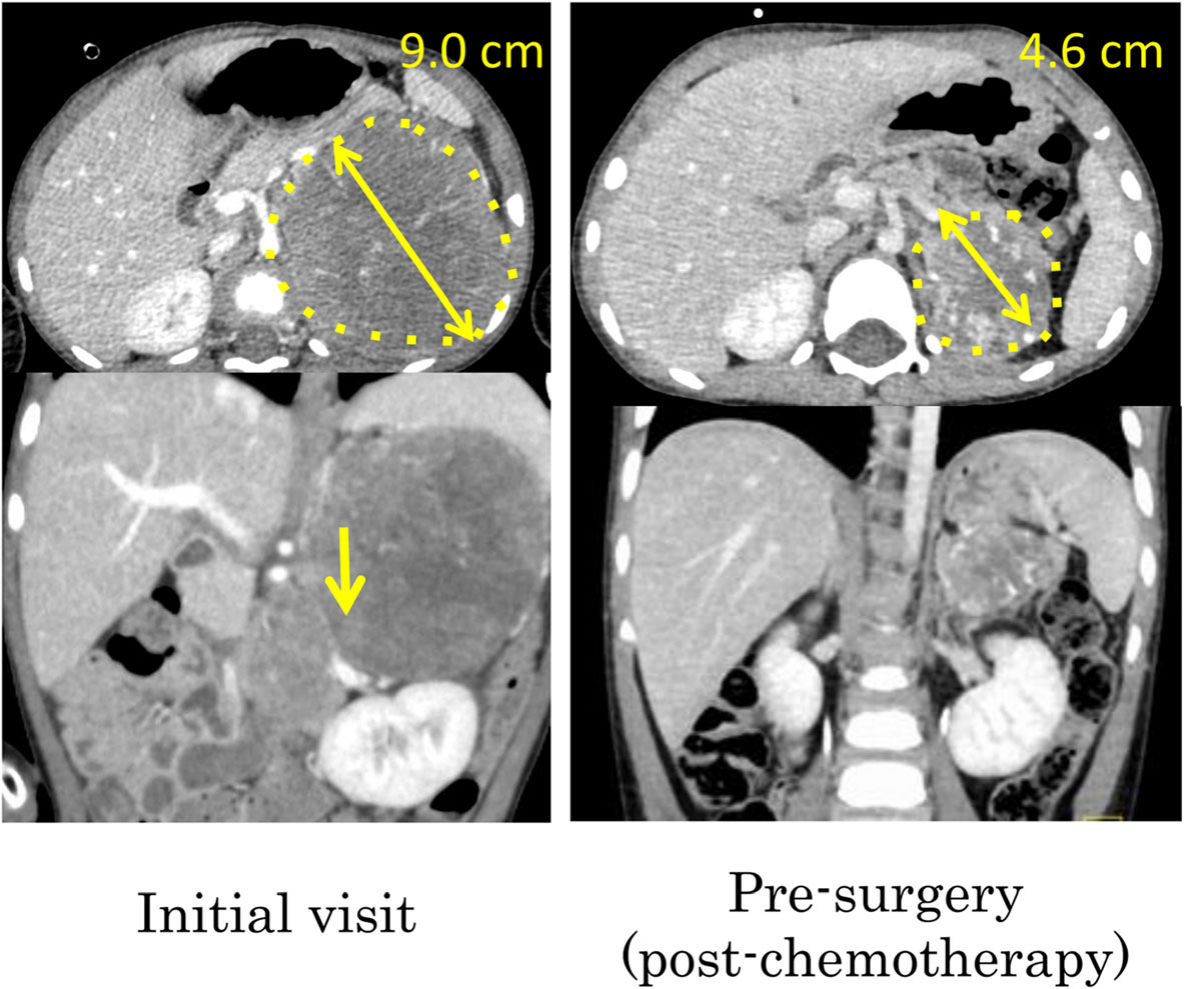
(Case 1): A 28-month-old boy (International Neuroblastoma Staging System [INSS]: stage 4, Children’s Oncology Group [COG] risk: high, surgical duration: 146 min, and blood loss: 53 mL). Total resection was laparoscopically performed. Outcome: progression-free survival, 24 months

**Fig. 4:**
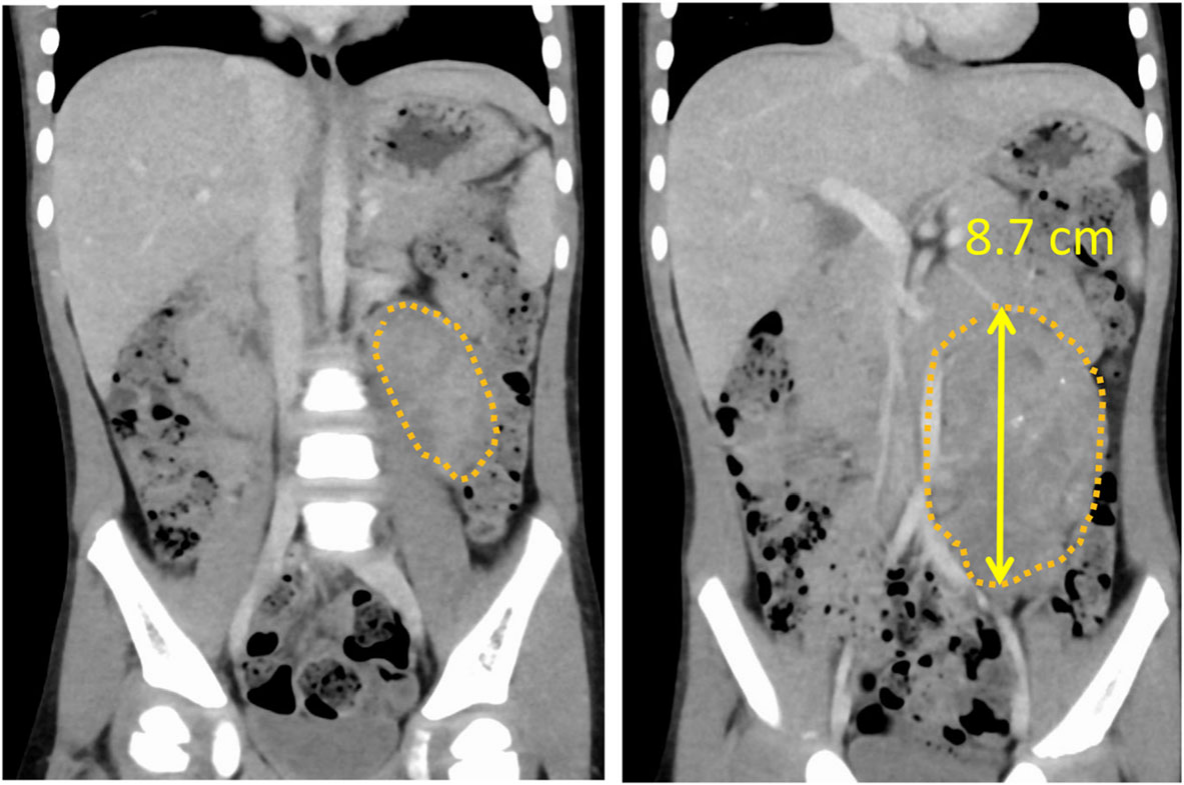
[Case 2 (pre-surgery)]: A 51-month-old boy (International Neuroblastoma Staging System [INSS]: stage 1, Children’s Oncology Group [COG] risk: low, surgical duration: 253 min, and blood loss: 61 mL). Total resection was laparoscopically performed. The tumor was in contact with the aorta, but renal vessels were intact. Outcome: progression-free survival, 16 months

Comparing conditions between the initial visit and pre-surgery (after neoadjuvant therapy) among the IDRF-negative patients, we found that the tumor size markedly decreased at pre-surgery (3.4 cm; range, 1.1–8.7; *p* = 0.031) compared with the initial visit (5.2 cm; range, 3.1–11.3). In addition, five patients who underwent laparoscopic resection transitioned from IDRF-positive to IDRF-negative after chemotherapy. For example, in Case 1 (a 28-month-old male at high risk), although the tumor was found to envelop the renal vessels at the initial visit, the tumor size markedly decreased (from 9.0 cm to 4.6 cm) and IDRFs were absent on imaging after chemotherapy (Fig. 3). This patient remained progression-free for 24 months after laparoscopic surgery. In Case 2 (a 51-month-old male at low risk), the tumor size was 8.7 cm and IDRFs were absent; thus, we performed laparoscopic total excision and removed the tumor through a Pfannenstiel incision without any complication (Fig. 4). This patient remained progression-free for 16 months after surgery.

## Discussion

The advantages of MIS compared with those of open surgery, include marked reduction in postoperative pain and surgical site infection, and cosmesis [19]. Accordingly, there were significant differences in the volume of blood loss and time required to initiate postoperative feeding between laparotomy and laparoscopy for IDRF-negative patients. Thus, we considered that laparoscopy was feasible for IDRF-negative patients. However, the tumor size limit for laparoscopic excision is unclear, and most reports have supported the indication of laparoscopic excision for tumors <4–6 cm [7, 20, 21]. In our laparoscopic IDRF-negative cases, the tumor sizes at operation were 1.1, 2.7, 2.8, 3.6, 4.6, 5.0 and 8.7 cm.

Because no obvious vascular involvement was present, we determined that the patient (Case 2) could undergo laparoscopic total resection and proceeded with the surgery. Five patients who underwent laparoscopic resection transitioned to IDRF-negative after chemotherapy. This finding indicates the importance of the timing of surgery in advanced cases. Simon et al. reported that 246 of 278 patients with stage 4 neuroblastoma aged 18 months or older did not undergo surgery prior to chemotherapy [22]. Kelleher et al. compared the clinical outcomes between laparotomy and laparoscopy, including advanced cases and found no difference in mortality among patients who met the selection criteria for the laparoscopic resection which were absence of vascular encasement and size <5 cm as the greatest dimension. Other patients underwent laparotomy [19, 23].

We performed neuroblastoma resection in 13 patients after introducing laparoscopic surgery, and of these, four underwent laparotomy (all were IDRF-positive) and nine underwent laparoscopy (two IDRF positive for contact with or encasement of renal vessels and seven IDRF negative). One IDRF-positive patient with encasement of renal vessels was converted from laparoscopy to laparotomy because dissection appeared difficult owing to strong adhesion with renal vessels. Partial resection under laparotomy was performed in this patient, and we had to carefully determine the indication for laparoscopy, considering the obvious vascular involvement in this case. The tumor in the other IDRF-positive patient made contact with the renal vessels and inferior vena cava; however, dissection was possible by laparoscopic surgery, and total tumor resection was macroscopically performed. Furthermore, a negative margin was histopathologically diagnosed. This patient currently remains progression-free for 23 months after high-dose chemotherapy and autologous peripheral blood stem cell transplantation. Although IDRF is positive if renal vessels are not only encased but also in contact with the tumor, in our experience, laparoscopic surgery could potentially be used for contacted cases [11]. Thus, we still considered IDRF in renal vessels as IDRF-negative. Irtan et al. reported that the completeness resection was related only to the number of preoperative IDRFs. They treated patients according to ongoing SIOPEN protocols, which included a high-risk group who received high-dose chemotherapy followed by stem cell rescue before surgery. They suggested IDRF assessment after neoadjuvant chemotherapy is useful in these patients [24].

Another advantage of laparoscopic surgery is a reduced risk of intestinal obstruction, which is critical for pediatric oncology patients, especially if they receive radiotherapy [25]. In our study, all patients in the laparoscopy group started oral feeding within 3 days after surgery and no patients presented symptoms of ileus. However, we had too few cases to prove the competitive advantage of laparoscopy over laparotomy.

The results of the present study showed no differences in short-term prognosis between these two procedures; thus, we believe that laparoscopic surgery can achieve favorable outcomes comparable to those of laparotomy in IDRF-negative patients, even those considered at high risk.

This study was limited by its small sample size and short observation period. Future studies involving more patients and a longer follow-up period are warranted. Moreover, because this was a single-center, retrospective study, a multicenter, prospective study would be ideal to assess the merits of minimally invasive surgery for this patient population.

This is the first report for the treatment of neuroblastoma comparing laparotomy and laparoscopy for the treatment of neuroblastoma without IDRFs.

## Conclusions

Although the limited number of cases and short-term observation period prevented the investigation of long-term outcomes, we found that laparoscopic surgery was a feasible minimally invasive procedure for the resection of neuroblastoma, particularly in IDRF-negative patients. Further studies focused on oncologic and safety outcomes are warranted to assess the merits of this surgical approach in neuroblastomas.

## Abbreviations

COG: Children’s Oncology Group
HVA: Homovanillic acid
IDRFs: Image-defined risk factors
INRG: International Neuroblastoma Risk Group
INSS: International Neuroblastoma Staging System
MIS: Minimally invasive surgery
MYCN: N-myc proto-oncogene protein
NSE: Neuron-specific enolase
VMA: Vanillylmandelic acid

## References

[1] Koivusalo AI, Pakarinen MP, Rintala RJ, Saarinen-Pihkala UM (2014). Surgical treatment of neuroblastoma: twenty-three years of experience at a single institution. Surg Today.

[2] Brodeur GM, Pritchard J, Berthold F, Carlsen NL, Castel V, Castelberry RP, De Bernardi B, Evans AE, Favrot M, Hedborg F (1993). Revisions of the international criteria for neuroblastoma diagnosis, staging, and response to treatment. J Clin Oncol Off J Am Soc Clin Oncol.

[3] Monclair T, Mosseri V, Cecchetto G, De Bernardi B, Michon J, Holmes K (2015). Influence of image-defined risk factors on the outcome of patients with localised neuroblastoma. A report from the LNESG1 study of the European International Society of Paediatric Oncology Neuroblastoma Group. Pediatr Blood Cancer.

[4] Cecchetto G, Mosseri V, De Bernardi B, Helardot P, Monclair T, Costa E, Horcher E, Neuenschwander S, Toma P, Rizzo A (2005). Surgical risk factors in primary surgery for localized neuroblastoma: the LNESG1 study of the European International Society of Pediatric Oncology Neuroblastoma Group. J Clin Oncol Off J Am Soc Clin Oncol.

[5] Brisse HJ, McCarville MB, Granata C, Krug KB, Wootton-Gorges SL, Kanegawa K, Giammarile F, Schmidt M, Shulkin BL, Matthay KK (2011). Guidelines for imaging and staging of neuroblastic tumors: consensus report from the International Neuroblastoma Risk Group Project. Radiology.

[6] de Lijster MS, Bergevoet RM, van Dalen EC, Michiels EMC, Caron HN, Kremer LCM, Aronson DC. Minimally invasive surgery versus open surgery for the treatment of solid abdominal and thoracic neoplasms in children. Cochrane Database Syst Rev. 2010;17(3):1–13.

[7] Iwanaka T, Kawashima H, Uchida H (2007). The laparoscopic approach of neuroblastoma. Semin Pediatr Surg.

[8] Iwanaka T, Arai M, Ito M, Kawashima H, Yamamoto K, Hanada R, Imaizumi S (2001). Surgical treatment for abdominal neuroblastoma in the laparoscopic era. Surg Endosc.

[9] Metzelder ML, Kuebler JF, Shimotakahara A, Glueer S, Grigull L, Ure BM (2007). Role of diagnostic and ablative minimally invasive surgery for pediatric malignancies. Cancer.

[10] Kim T, Kim DY, Cho MJ, Kim SC, Seo JJ, Kim IK (2011). Use of laparoscopic surgical resection for pediatric malignant solid tumors: a case series. Surg Endosc.

[11] Irtan S, Brisse HJ, Minard-Colin V, Schleiermacher G, Canale S, Sarnacki S. Minimally invasive surgery of neuroblastic tumors in children: Indications depend on anatomical location and image-defined risk factors. Pediatr Blood Cancer. 2015;62(2):257–61. 10.1002/pbc.25248 25284263

[12] Mattioli G, Avanzini S, Prato AP, Pio L, Granata C, Garaventa A, Conte M, Manzitti C, Montobbio G, Buffa P (2014). Laparoscopic resection of adrenal neuroblastoma without image-defined risk factors: a prospective study on 21 consecutive pediatric patients. Pediatr Surg Int.

[13] Heloury Y, Muthucumaru M, Panabokke G, Cheng W, Kimber C, Leclair MD (2012). Minimally invasive adrenalectomy in children. J Pediatr Surg.

[14] Tsuchida Y, Miyauchi J, Kuroiwa M, Suzuki N, Sakamoto J, Suzuki M, Shitara T (2005). Histologic survey of neuroblastomas after intensive induction chemotherapy. Pediatr Blood Cancer.

[15] Kaneko M, Tsuchida Y, Uchino J, Takeda T, Iwafuchi M, Ohnuma N, Mugishima H, Yokoyama J, Nishihira H, Nakada K (1999). Treatment results of advanced neuroblastoma with the first Japanese study group protocol. Study Group of Japan for Treatment of Advanced Neuroblastoma. J Pediatr Hematol Oncol.

[16] Kaneko M, Nishihira H, Mugishima H, Ohnuma N, Nakada K, Kawa K, Fukuzawa M, Suita S, Sera Y, Tsuchida Y (1998). Stratification of treatment of stage 4 neuroblastoma patients based on N-myc amplification status. Study Group of Japan for Treatment of Advanced Neuroblastoma, Tokyo, Japan. Med Pediatr Oncol.

[17] Amano H, Uchida H, Kawashima H, Deie K, Murase N, Makita S, Yokota K, Tanaka Y (2015). The umbilical Benz incision for reduced port surgery in pediatric patients. JSLS.

[18] Dindo D, Demartines N, Clavien PA (2004). Classification of surgical complications: a new proposal with evaluation in a cohort of 6336 patients and results of a survey. Ann Surg.

[19] Blinman T, Ponsky T (2012). Pediatric minimally invasive surgery: laparoscopy and thoracoscopy in infants and children. Pediatrics.

[20] Castilho LN, Castillo OA, Denes FT, Mitre AI, Arap S (2002). Laparoscopic adrenal surgery in children. J Urol.

[21] Simon T, Haberle B, Hero B, von Schweinitz D, Berthold F (2013). Role of surgery in the treatment of patients with stage 4 neuroblastoma age 18 months or older at diagnosis. J Clin Oncol Off J Am Soc Clin Oncol.

[22] de Lagausie P, Berrebi D, Michon J, Philippe-Chomette P, El Ghoneimi A, Garel C, Brisse H, Peuchmaur M, Aigrain Y (2003). Laparoscopic adrenal surgery for neuroblastomas in children. J Urol.

[23] Kelleher CM, Smithson L, Nguyen LL, Casadiego G, Nasr A, Irwin MS, Gerstle JT (2013). Clinical outcomes in children with adrenal neuroblastoma undergoing open versus laparoscopic adrenalectomy. J Pediatr Surg.

[24] Irtan S, Brisse HJ, Minard-Colin V, Schleiermacher G, Galmiche-Rolland L, Le Cossec C, Elie C, Canale S, Michon J, Valteau-Couanet D (2015). Image-defined risk factor assessment of neurogenic tumors after neoadjuvant chemotherapy is useful for predicting intra-operative risk factors and the completeness of resection. Pediatr Blood Cancer.

[25] Madenci AL, Fisher S, Diller LR, Goldsby RE, Leisenring WM, Oeffinger KC, Robison LL, Sklar CA, Stovall M, Weathers RE (2015). Intestinal obstruction in survivors of childhood cancer: a report from the childhood cancer survivor study. J Clin Oncol Off J Am Soc Clin Oncol.

